# Bivariate Genome-Wide Association Analyses Identified Genes with Pleiotropic Effects for Femoral Neck Bone Geometry and Age at Menarche

**DOI:** 10.1371/journal.pone.0060362

**Published:** 2013-04-04

**Authors:** Shu Ran, Yu-Fang Pei, Yong-Jun Liu, Lei Zhang, Ying-Ying Han, Rong Hai, Qing Tian, Yong Lin, Tie-Lin Yang, Yan-Fang Guo, Hui Shen, Inderpal S. Thethi, Xue-Zhen Zhu, Hong-Wen Deng

**Affiliations:** 1 School of Public Health and Tropical Medicine, Tulane University, New Orleans, Louisiana, United States of America; 2 Center of System Biomedical Sciences, School of Medical Instrument and Food Engineering, University of Shanghai for Science and Technology, Shanghai, P. R. China; 3 Inner Mongolia People’s Hospital, Hohhot, P. R. China; 4 School of Life Science and Technology, Xi’an Jiaotong University, Xi’an, Shanxi, P. R. China; 5 School of Basic Medical Science, Institute of Bioinformatics, Southern Medical University, Guangzhou, Guangdong, P. R. China; Harvard Medical School, United States of America

## Abstract

Femoral neck geometric parameters (FNGPs), which include cortical thickness (CT), periosteal diameter (W), buckling ratio (BR), cross-sectional area (CSA), and section modulus (Z), contribute to bone strength and may predict hip fracture risk. Age at menarche (AAM) is an important risk factor for osteoporosis and bone fractures in women. Some FNGPs are genetically correlated with AAM. In this study, we performed a bivariate genome-wide association study (GWAS) to identify new candidate genes responsible for both FNGPs and AAM. In the discovery stage, we tested 760,794 SNPs in 1,728 unrelated Caucasian subject, followed by replication analyses in independent samples of US Caucasians (with 501 subjects) and Chinese (with 826 subjects). We found six SNPs that were associated with FNGPs and AAM. These SNPs are located in three genes (i.e. NRCAM, IDS and LOC148145), suggesting these three genes may co-regulate FNGPs and AAM. Our findings may help improve the understanding of genetic architecture and pathophysiological mechanisms underlying both osteoporosis and AAM.

## Introduction

Bone strength at the hip is directly related to the risk of hip fracture, the most serious and disabling type of osteoporotic fractures [1]. Femoral neck geometry is a major determinant of the mechanical resistance of the hip and plays an important role, independent of bone mineral density (BMD), in determining bone strength and osteoporotic fractures [2]. Femoral neck geometric parameters (FNGPs) measure bone structural properties (such as shape, size, and microarchitecture) and are believed to be as good as BMD in predicting hip fracture risk [3]. FNGPs, including cortical thickness (CT), periosteal diameter (W), buckling ratio (BR), cross-sectional area (CSA), and section modulus (Z), can be conveniently and accurately inferred from dual energy X-ray absorptiometry (DXA) measurements.

FNGPs have strong genetic determination with heritability ranging from 40–50% [4]–[7]. Earlier studies, including ours in Caucasians and Chinese, have identified some promising candidate genes associated with FNGPs [8]–[12].

Menarche is the first menstrual cycle in female human beings, which occurs when thickened endometrial tissue undergoes a sudden death because of fluctuations of estrogen levels. Age at menarche (AAM) is an important factor that affects women’s health. Late AAM is related with higher risk of osteoporosis in women, which may partially due to the less exposure to estrogen [13], [14]. It has been shown that AAM is under strong genetic control and heritability of AAM is as high as 50–70% [15], [16].

Since FNGPs and AAM are heritable traits highly related to risk of osteoporosis and women’s health, it would be interesting to investigate whether there are pleiotropic genes that influence variation in both FNGPs and AAM. Previous studies were largely conducted on FNGPs or AAM separately using univariate analysis method which does not consider potential correlations between them [7], [17]. This problem could be addressed by performing multivariate analysis which analyzes correlated traits simultaneously. Compared to univariate analysis, multivariate analysis has an advantage in detecting pleiotropic genes by considering the correlations between traits in the model. In addition, the multiple testing problems caused by testing different traits separately in univariate analysis can be alleviated in multivariate analysis.

Here we report the first bivariate genome-wide association study (GWAS) for FNGPs and AAM in a sample of 1,728 unrelated US Caucasian female subjects followed by replication analyses in independent Caucasians and Chinese samples. We identified three genes (i.e. NRCAM, IDS and LOC148145) that were associated with both FNGPs and AAM, suggesting their roles in co-regulating FNGPs and AAM. Our findings may help improve the understanding of genetic architecture and pathophysiological mechanisms underlying both osteoporosis and AAM.

## Methods

### Subjects

The discovery sample contained 1,728 unrelated Caucasian female subjects recruited in Midwestern US (Kansas City, MO, and Omaha, NE) for studies of osteoporosis and related health problems. All the identified subjects were US Caucasians of European origin. The replication samples included Caucasians and Chinese. The Caucasian replication sample included 501 unrelated female subjects living in Omaha, NE, USA, and its surrounding areas. There was no overlap between the discovery and replication Caucasian samples. The Chinese replication sample included 826 unrelated female subjects living in Changsha or Xi’an, China.

The study was approved by Institutional Review Boards of Creighton University, University of Missouri-Kansas City, Hunan Normal University of China and Xi’an Jiaotong University of China. All the subjects signed informed-consent documents and completed structured questionnaires. The exclusion criteria were detailed in our previous publications [18]. Briefly, subjects with chronic diseases and conditions that might affect bone metabolism and AAM were excluded from this study.

Areal BMD (g/cm^2^) and region area (cm^2^) of FN were measured using dual-energy X-ray absorptiometry scanners Hologic QDR 4500 W (Hologic Inc., Bedford, MA, USA). The machines were calibrated daily. The coefficient of variation (CV) values obtained from the DXA measurements for FN bone size and FN BMD were 1.94% and 1.87%. Bone geometric parameters were calculated using the DXA-derived FN BMD and bone size. The methods for calculating these variables were detailed elsewhere [7], [19], [20]. The five estimated FNGPs are CT, W, BR, CSA, and Z. CT is an estimate of mean cortical thickness, W is the outer diameter of the bone, BR is an index of cortical instability indicating the risk of fracture by buckling, CSA is an indicator of bone axial compression strength, and Z is an index of bone bending strength indicating the bending resistance of a tube.

### Genotyping

For each study subject, we extracted genomic DNA from peripheral blood leukocytes using standard protocols, and genotyping experiments were performed strictly following the standard protocol recommended by related manufacturer. The subjects of the discovery sample and the Chinese replication sample were genotyped using Affymetrix Genome-wide Human SNP 6.0 genotyping arrays (Affymetrix, Santa Clara, CA, USA) which include 909,622 SNPs [21]. The Caucasian replication sample was genotyped using Affymetrix Human Mapping 500 K arrays which included 500,567 SNPs.

### Quality Control

In order to obtain high quality genotyping data, we followed strict quality control procedures. Samples with a minimum call rate of 95% were included in the analyses. For the Caucasian sample, the final mean call rate reached a level of 98.93%. We discarded SNPs that deviated from Hardy-Weinberg equilibrium (*p*<0.0001) and those with a minor allele frequency (MAF) <0.01. After quality control, the numbers of SNPs available for association analysis were 760,794 in the discovery sample, 702,413 in the Chinese replication sample, and 407,192 in the Caucasian replication sample.

### Statistical Analyses

The five FNGPs were adjusted by age, height and weight [22], while AAM was adjusted by height and weight [23], [24] using Minitab (Minitab Inc., State College, PA, USA). The covariates-adjusted phenotypic values were used in subsequent association analyses. We tested the phenotypic correlation between FNGPs and AAM using bivariate correlation analysis in Minitab.

The association analyses between genotype and the covariates-adjusted traits were performed using a bivariate linear regression model. The model is represented by 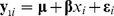, where ***y*** *_i_* is the vector of the two traits for individual *i*; ***μ*** is the vector of grand means; ***β*** is the vector of its effects, and *x_i_* is the genotype score for individual *i*. ***ε*** *_i_* is the vector of residues following a multivariate normal distribution with mean zero. The genotype *x_i_* was encoded with an additive mode of inheritance. The association was examined by testing the significance of ***β***, and the test was performed with R package *lm*. Individual *p* values achieved in the three studied samples were then combined by fisher’s method [25]. Genomic control approach was used to evaluate population effect and control potential population stratification that may lead to spurious association results. The *p* value of 5×10^−8^ was used as the threshold to claim significant associations at the genome-wide level, after accounting for multiple-testing by applying Bonferroni correction. Since Bonferroni correction is quite conservative, we reported SNPs that achieved a *p* value of 10^−5^ or less in the discovery stage. For replication, due to the prior evidence of association, we used a threshold of the nominal *p* level of 0.05.

We evaluated the proportion of phenotype correlations between each trait pairs explained by the reported SNPs. We define corr1 as the original phenotype correlation coefficients and corr2 as the phenotype correlation coefficients after adjusted by the SNPs. The proportion of correlations between each trait pairs explained by the reported SNPs (corrp) was calculated by the formula: 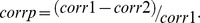


For the SNPs showing potential pleiotropic effects, we further investigated the effect direction and causal relationship. The effect direction of the SNPs was evaluated using a linear regression model implemented in PLINK [26]. The causal relationship of the SNPs was examined by comparing adjusted/conditional models in bivariate linear regression analyses, in which the genotype of each of the reported SNPs was adjusted as a covariate in turn.

For comparison purpose, we also performed univariate association analyses for each trait using a univariate linear regression model with the R package *lm*. To compare statistical power between univariate and bivariate association analyses, we performed power analyses using the *GEE* (Generalized Estimation Equation) implemented in R. The power analyses were based on the sample sizes of 1,728, 826, and 501 unrelated subjects as used in the present study for discovery and replication analyses. Simulation analysis was performed to calculate the power.

The coefficient of linkage disequilibrium (LD) between specific SNPs was obtained from the Haploview system [27]. We used the FASTSNP program (http://fastsnp.ibms.sinica.edu.tw) to explore potential functions of the reported SNPs [28].

## Results

The basic characteristics of the discovery and replication samples are summarized in Table 1. Correlation analysis of the study traits showed that AAM was significantly correlated with FNGPs. In the discovery sample, significant correlations were observed between AAM and three FNGPs (CT, W, and BR), and the correlation coefficients were −0.054 (*p* = 0.028) for AAM and CT, 0.043 (*p* = 0.082) for AAM and W, 0.077 (*p* = 0.002) for AAM and BR, respectively. The significant correlations observed here are consistent with previous findings of others [29], [30]. We subsequently focused biviarate analyses on AAM and these three FNGPs.

**Table 1:** Basic characteristics of the study subjects.

Traits	Discovery Caucasians(n = 1728)	Replication Caucasians(n = 501)	Replication Chinese(n = 826)
Age (years)	51.58 (12.92)	50.15 (17.69)	37.46 (13.77)
Height (cm)	163.28 (6.27)	163.85 (6.51)	158.38 (5.22)
Weight (kg)	71.45 (16.04)	71.32 (15.92)	54.63 (8.09)
AAM (years)	12.92 (1.58)	12.90 (1.49)	13.91 (1.61)
CT (cm)	0.15 (0.02)	0.15 (0.03)	0.14 (0.02)
W (cm)	3.30 (0.34)	3.36 (0.25)	3.11 (0.35)
BR	11.46 (2.42)	11.61 (2.74)	11.07 (2.76)
CSA (cm^2^)	2.45 (0.48)	2.48 (0.45)	2.24 (0.38)
Z (cm^3^)	1.45 (0.36)	1.49 (0.31)	1.26 (0.29)

Note:

All the values are presented as mean (standard deviation).

We identified six SNPs that were associated with both FNGPs and AAM in the discovery sample (*p*<10^−5^). These SNPs were replicated in independent Caucasian and/or Chinese replication samples (Table 2). Among them, rs4141232 is located in the upstream of the LOC148145 gene (*p* = 4.12×10^−7^ for AAM-CT), rs6975557 and rs13230316 are located in the intron of the NRCAM (neuronal cell adhesion molecule isoform A) gene (*p* = 2.82×10^−5^ and 5.58×10^−5^ for AAM-W, respectively), and the other three SNPs, rs5980450, rs4844014, and rs7064959, are located in the downstream of the IDS (Iduronate-2-sulfatase) gene (*p* = 6.76×10^−5^, 7.31×10^−5^ and 8.64×10^−5^ for AAM-W, respectively). Three SNPs (rs4141232, rs6975557 and rs13230316) were replicated in the Caucasian sample (*p = *0.03, 8.33×10^−5^ and 1.10×10^−4^, respectively). The other three SNPs (rs5980450, rs4844014, and rs7064959) near the IDS gene were replicated in the Chinese sample (*p = *0.05) (Table 2). Combined *p* values of meta-analyses are also shown in Table 2.

**Table 2:** Results of bivariate GWAS for AAM and three FNGPs (*p*<10^−5^ in the discovery sample).

Traits pair	SNP	Chr	Position	Gene	*P* valuein discovery sample	*P* valuein replication Caucasian	*P* valuein replication Chinese	Combined*p* value^1^	Combined*p* value^2^
AAM-CT									
	rs8113142	19	33704761	LOC148145	3.87×10^−7^	–	0.73	–	4.54×10^−6^
	**rs4141232**	**19**	**33727396**	**LOC148145**	**4.12**×**10** ^−**7**^	**0.03**	0.89	2.37×10^−7^	5.80×10^−6^
	rs4805257	19	33822305	LOC148145	1.62×10^−6^	–	0.58	–	1.40×10^−5^
	rs6578985	11	2094715	IGF2	1.06×10^−6^	0.36	0.18	6.02×10^−6^	3.14×10^−6^
	rs6578987	11	2098162	IGF2	1.28×10^−6^	–	0.14	–	2.96×10^−6^
	rs6578986	11	2094728	IGF2	1.28×10^−6^	–	0.34	–	6.81×10^−6^
	rs4929957	11	2084885	IGF2	4.97×10^−6^	–	0.08	–	6.26×10^−6^
AAM-W									
	rs7929583	11	86904123	RAB38	4.35×10^−6^	0.85	0.85	5.00×10^−5^	5.00×10^−5^
	rs12146626	11	86907172	RAB38	4.25×10^−6^	0.92	0.84	5.26×10^−5^	4.83×10^−5^
	rs10898723	11	86904392	RAB38	8.58×10^−6^	0.84	0.87	9.25×10^−5^	9.56×10^−5^
	**rs6975557**	**7**	**107760533**	**NRCAM**	**2.82**×**10** ^−**5**^	**8.33**×**10** ^−**5**^	0.45	**4.84**×**10** ^−**8**^	1.56×10^−4^
	**rs13230316**	**7**	**107778803**	**NRCAM**	**5.58**×**10** ^−**5**^	**1.10**×**10** ^−**4**^	0.58	1.22×10^−7^	3.67×10^−4^
	**rs5980450**	**X**	**148285691**	**IDS**	**6.76**×**10** ^−**5**^	–	**0.05**	–	4.60×10^−5^
	**rs4844014**	**X**	**148212344**	**IDS**	**7.31**×**10** ^−**5**^	0.39	**0.05**	3.27×10^−4^	4.94×10^−5^
	**rs7064959**	**X**	**148294515**	**IDS**	**8.64**×**10** ^−**5**^	–	**0.05**	–	5.77×10^−5^
AAM-BR									
	rs4141232	19	33727396	LOC148145	6.99×10^−7^	0.96	0.87	1.02×10^−5^	9.31×10^−6^
	rs7929583	11	86904123	RAB38	3.54×10^−6^	0.84	0.58	4.08×10^−5^	2.89×10^−5^
	rs12146626	11	86907172	RAB38	4.36×10^−6^	0.44	0.58	2.72×10^−5^	3.51×10^−5^
	rs10898723	11	86904392	RAB38	7.43×10^−6^	0.35	0.62	3.69×10^−5^	6.12×10^−5^

Note:

Combined *p* value^1^: Combined *p* values by joint analyses of the Caucasian discovery and the Caucasian replication samples.

Combined *p* value^2^: Combined *p* values by joint analyses of the Caucasian discovery and the Chinese replication samples.

**−**: *p* value not available.

Bold: SNPs that were replicated in the replication samples.

The characteristics of the six SNPs bivariately associated with FNGPs and AAM are shown in Table 3. The proportions of phenotype correlation explained by the six SNPs were 18.52% for AAM-CT, 25.26% for AAM-W, and 12.99% for AAM-BR, respectively (Table 4).

**Table 3:** Characteristics of SNPs bivariately associated with FNGPs and AAM.

SNP	Chr	Position	Gene	Role	Allele^a^	MAF^b^	MAF^c^	MAF^d^	MAF^e^	MAF^f^	AAM-CT	AAM-W	AAM-BR
rs4141232	19	33727396	LOC148145	upstream	C/T	0.16	0.15	0.18	0.20	0.23	4.12×10^−7^	1.09×10^−3^	6.99×10^−7^
rs6975557	7	107760533	NRCAM	intron	G/A	0.28	0.26	0.26	0.45	0.41	6.29×10^−3^	2.82×10^−5^	5.31×10^−4^
rs13230316	7	107778803	NRCAM	intron	G/C	0.27	0.26	0.25	0.45	0.38	7.14×10^−3^	5.58×10^−5^	6.63×10^−4^
rs5980450	X	148285691	IDS	downstream	A/G	0.06	–	0.03	0.15	0.15	0.44	6.76×10^−5^	6.07×10^−3^
rs4844014	X	148212344	IDS	downstream	C/A	0.06	0.06	0.03	0.14	0.15	0.55	7.31×10^−5^	6.91×10^−3^
rs7064959	X	148294515	IDS	downstream	G/A	0.06	–	0.04	0.13	0.15	0.75	8.64×10^−5^	0.01

Note:

a The first allele represents the minor allele of each locus.

b Minor allele frequency calculated in our discovery Caucasian sample (n = 1728).

c Minor allele frequency calculated in our replication Caucasian sample (n = 501).

d Minor allele frequency reported for Caucasians in the public database of HapMap CEU.

e Minor allele frequency calculated in our replication Chinese subjects.

f Minor allele frequency reported for Chinese in the public database of HapMap.

**–**: MAF not available.

**Table 4:** Proportions of phenotype correlation explain correlations coefficients of three trait pairs.

	AAM-CT	AAM-W	AAM-BR
corr1	**−**0.05	0.04	0.08
corr2	**−**0.04	0.03	0.07
corrp	18.52%	25.26%	12.99%

Note:

Corr1: The original phenotype correlation coefficients.

Corr2: The phenotype correlation coefficients after adjusted by the SNPs.

Corrp: The proportion of correlations between each trait pairs explained by the reported SNPs, which is calculated by 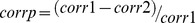
_._

Based on SNPs genotyped in GWAS, we estimated inflation factor (λ) which is a measure of population stratification. Generally, for a homogenous population with no stratification the value of λ should be equal or close to 1. In our GWAS cohorts, the estimated λ values for AAM, CT, W, and BR were 0.938, 0.982, 0.934, and 0.936, respectively, suggesting no or very modest population stratification, if any.

**Figure 1:**
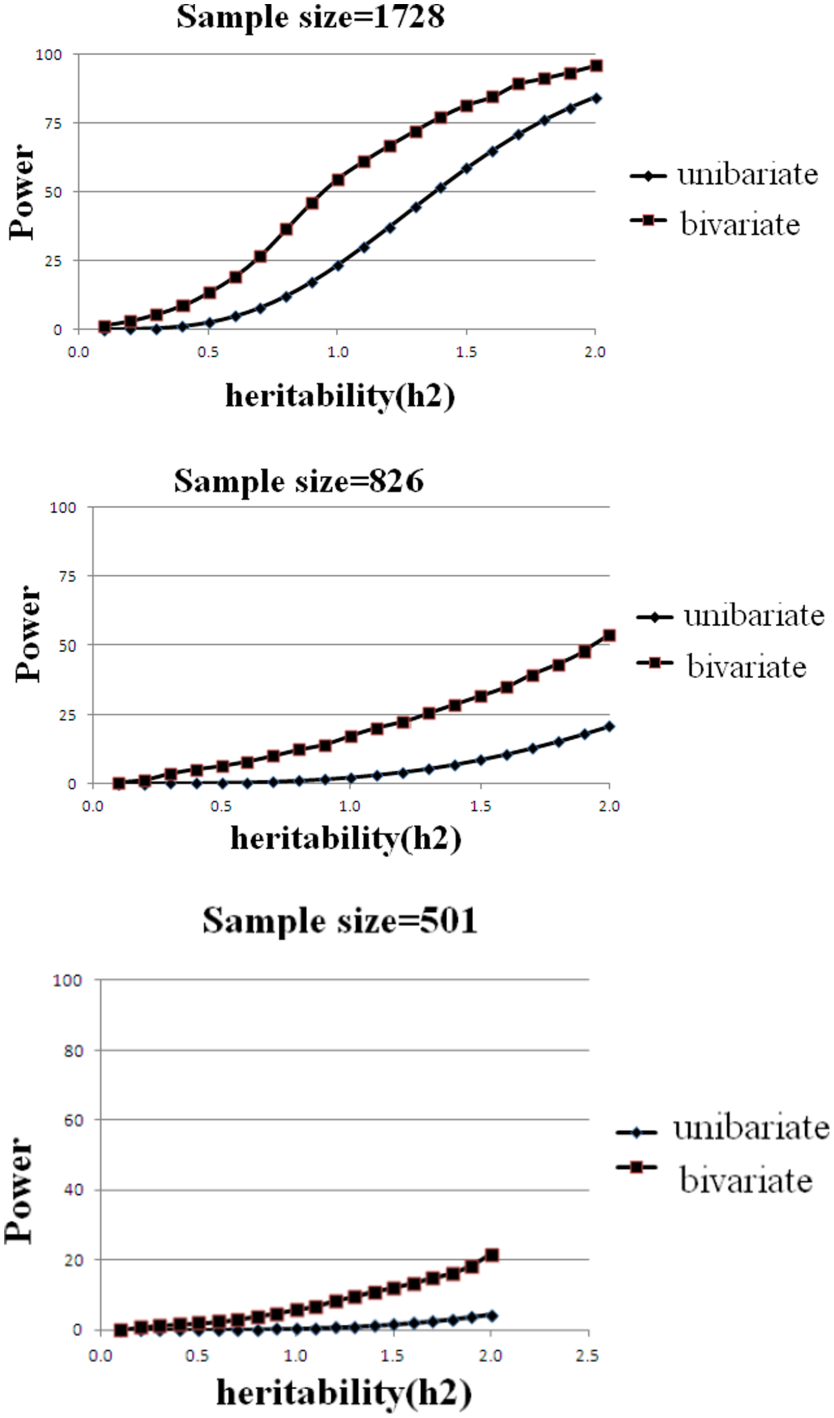
Comparison of statistical power of bivariate vs. univariate association analyses in three samples of this study. The Y axis shows the expected power, and the X axis shows the heritability. Type 1 error was set at 0.05 and minor allele frequency (MAF) was set at 0.1. The sample sizes were 1728 (discovery Caucasians), 826 (replication Chinese) and 501 (replication Caucasians), respectively.

We performed univariate association analyses for the six identified SNPs in the three studied samples. As presented in Table 5, *p* values of univariate association analyses were less significant than those of bivariate analyses. Power calculation showed that bivariate analysis exhibited consistently higher statistical power than univariate analysis did for any of the three samples (Fig. 1).

**Table 5:** Results of univariate association analyses for the six SNPs in the discovery sample and the replication samples.

SNP	Univariate *p* valuediscovery sample (n = 1728)				Univariate *p* valueReplication Chinese sample (n = 826)				Univariate *p* valueReplication Caucasian sample (n = 501)			
	AAM	CT	W	BR	AAM	CT	W	BR	AAM	CT	W	BR
rs4141232	3.48×10^−3^	7.70×10^−6^	6.67×10^−3^	5.88×10^−6^	0.82	0.95	0.63	0.81	0.46	0.01	0.96	0.66
rs6975557	0.02	0.03	8.62×10^−5^	7.57×10^−4^	0.28	7.57×10^−4^	0.53	7.57×10^−4^	0.63	0.66	2.09×10^−5^	0.57
rs13230316	0.03	0.02	1.25×10^−4^	6.30×10^−4^	0.36	6.30×10^−4^	0.65	6.30×10^−4^	0.75	0.59	2.49×10^−5^	0.68
rs5980450	0.47	0.40	2.36×10^−5^	3.70×10^−3^	0.02	3.70×10^−3^	0.39	3.70×10^−3^	**–**	**–**	**–**	**–**
rs4844014	0.77	0.39	1.91×10^−5^	3.13×10^−3^	0.02	3.13×10^−3^	0.43	3.13×10^−3^	0.17	0.40	0.93	0.91
rs7064959	0.86	0.51	1.79×10^−5^	5.00×10^−3^	0.03	0.42	0.33	0.32	**–**	**–**	**–**	**–**

Note:

**–**: *p* value not available.

The effect directions of these SNPs are presented in Table 6. A positive beta value means that the minor allele is associated with a higher trait value. Since these SNPs were not included in the SNP arrays scanned in the Caucasian replication sample, their effect directions are not available for this sample. The effect direction for AAM was contrary between discovery and Chinese replication samples. For CT, W and BR, two SNPs of the NRCAM gene had the same effect direction in both samples, while three SNPs near the IDS gene had contrary effect directions between the two samples.

**Table 6:** The effect direction of the SNPs in discovery and replication samples.

	Gene	Discovery Caucasian sample(n = 1728)					Replication Chinese sample (n = 826)			
		β/AAM	β/CT	β/W	β/BR	Minor allele	β/AAM	β/CT	β/W	β/BR
rs4141232	LOC148145	**−**0.13	0.20	**−**0.13	**−**0.21	C	0.02	**−**0.004	0.02	0.01
rs6975557	NRCAM	0.10	**−**0.09	0.16	0.13	G	**−**0.05	**−**0.04	0.03	0.07
rs13230316	NRCAM	0.09	**−**0.09	0.16	0.13	G	**−**0.03	**−**0.04	0.02	0.06
rs5980450	IDS	**−**0.07	**−**0.06	0.30	0.21	A	0.21	0.04	**−**0.06	**−**0.07
rs4844014	IDS	**−**0.06	**−**0.06	0.30	0.22	C	0.19	0.03	**−**0.05	**−**0.06
rs7064959	IDS	**−**0.03	**−**0.05	0.32	0.21	G	0.21	0.06	**−**0.06	**−**0.07

Note:

The effect direction was assessed using a linear regression model. A positive/negative regression coefficients (*β*) value means that the minor allele is associated with a higher/lower trait value. The three FNGP phenotypes were adjusted by age, height and weight while AAM was adjusted by height and weight.

We examined the causal relationships of the six SNPs (Table 7). It can be seen that for the SNPs located in the same gene, using the genotype of one SNP as a covariate, the association signals disappeared, suggesting these SNPs are in linkage disequilibrium. When the genotypes of the SNPs located in different genes were used as a covariate, the association signals remained, suggesting they are independent. From biology point of view, the concept of causality is more complex than comparison of adjusted/conditional models as used here. The causal effects of the variants need to be further explored and validated via deep re-sequencing of the gene locus and subsequent molecular functional studies.

**Table 7:** The causal relationship of the six SNPs for three trait pairs in the discovery sample.

	Covariate	Gene	rs4141232	rs6975557	rs13230316	rs5980450	rs4844014	rs7064959
AAM-CT								
	rs4141232	LOC148145	–	4.67×10^−3^	4.63×10^−3^	0.54	0.67	0.85
	rs6975557	NRCAM	4.26×10^−7^	–	0.96	0.55	0.64	0.77
	rs13230316	NRCAM	2.90×10^−7^	0.91	–	0.56	0.41	0.64
	rs5980450	IDS	8.10×10^−7^	9.09×10^−3^	8.89×10^−3^	–	0.84	0.83
	rs4844014	IDS	1.02×10^−6^	7.95×10^−3^	9.05×10^−3^	0.88	–	0.98
	rs7064959	IDS	1.38×10^−6^	8.69×10^−3^	9.90×10^−3^	0.91	0.99	–
AAM-W								
	rs4141232	LOC148145	–	2.43×10^−5^	4.39×10^−5^	9.03×10^−5^	9.33×10^−5^	1.30×10^−4^
	rs6975557	NRCAM	4.04×10^−4^	–	1.00	7.64×10^−5^	5.60×10^−5^	5.87×10^−5^
	rs13230316	NRCAM	2.76×10^−4^	0.94	–	6.98×10^−5^	8.78×10^−5^	9.29×10^−5^
	rs5980450	IDS	7.82×10^−4^	4.83×10^−5^	1.02×10^−4^	–	0.82	0.81
	rs4844014	IDS	7.76×10^−4^	5.00×10^−5^	1.15×10^−4^	0.86	–	0.91
	rs7064959	IDS	1.20×10^−3^	4.57×10^−5^	1.07×10^−4^	0.88	0.90	–
AAM-BR								
	rs4141232	LOC148145	–	2.58×10^−4^	2.81×10^−4^	8.40×10^−3^	8.94×10^−3^	0.02
	rs6975557	NRCAM	3.66×10^−7^	–	0.97	7.67×10^−3^	6.17×10^−3^	0.01
	rs13230316	NRCAM	2.74×10^−7^	0.92	–	6.42×10^−3^	2.43×10^−3^	6.94×10^−3^
	rs5980450	IDS	1.21×10^−6^	8.43×10^−4^	9.51×10^−4^	–	0.77	0.83
	rs4844014	IDS	1.34×10^−6^	7.92×10^−4^	1.07×10^−3^	0.83	–	0.97
	rs7064959	IDS	2.37×10^−6^	7.78×10^−4^	1.06×10^−3^	0.88	0.93	–

Note:

The causal relationship of the SNPs was examined by comparing adjusted/conditional models in bivariate linear regression analyses, in which the genotype of each of the six SNPs was adjusted as a covariate in turn. *P* values of the analyses are shown in the table.

**Figure 2:**
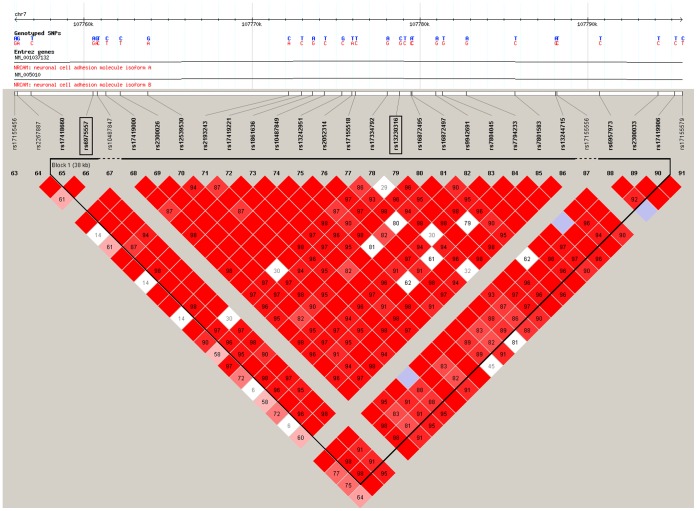
LD structure of the NRCAM gene in the discovery Caucasian sample. The region demarcated in red indicates that r^2^>0.9. The region includes only one LD block as indicated by triangle with black lines. SNP rs6975557 and rs13230316 are located within one block.

**Figure 3:**
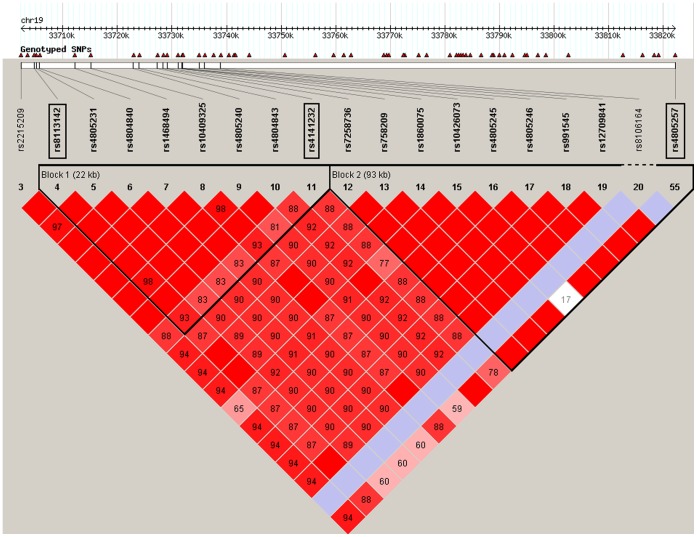
LD structure of the upstream of LOC148145 gene in the discovery Caucasian sample. The region demarcated in red indicates that r^2^>0.9. The region includes two LD blocks (Block1 and Block2) marked by triangles with black lines. SNPs rs8113142 and rs4141232 are located in Block1, and the SNP rs4805257 is located in Block 2.

Analysis using the software Haploview in the Caucasian sample showed that SNPs rs6975557 and rs13230316 located in the NRCAM gene are in the same LD block (r^2^ ≥ 0.97) (Fig. 2). The SNPs rs8113142, rs4141232, and rs4805257 near the LOC148145 gene are in two LD blocks (r^2^ = 0.93, and 0.88, respectively) (Fig. 3). The SNPs rs5980450, rs4844014, and rs7064959 near the IDS gene are not available in the Haploview.

Using the FASTSNP program, we investigated the potential functions of these six SNPs. The SNPs rs6975557 and rs13230316 are located at potential transcription factor-binding sites and thus may have a role in transcription regulation. A G→A change at rs6975557 may result in the elimination of the binding sites for transcription factor GATA-1, while a G→C change at rs13230316 may produce a change in the binding sites of S8 and OCT-1. The other four SNPs (i.e., rs4141232, rs5980450, rs4844014, and rs7064959) did not show known functions according to the FASTSNP program.

## Discussion

To the best of our knowledge, this is the first bivariate GWAS for FNGPs and AAM, which identified three novel genes (i.e., NRCAM, IDS and LOC148145) which may contribute to co-variation of FNGPs and AAM. GWAS were largely performed by analyzing individual traits separately in a univariate framework. Although univariate analysis is effective for discovering novel genes responsible for a specific disease or trait, the approach generally ignores the potential genetic co-predisposition to human diseases. Bivariate analysis considers the correlation between traits and has an advantage in identifying genes with pleiotropic effects. The approach may help reveal the interconnected pathophysiological networks for a spectrum of common human diseases [31], [32].

The gene NRCAM encodes a neuronal cell adhesion molecule [33]. Early studies showed that NRCAM gene expression increased during osteogenic and chondrogenic differentiation [34], suggesting it may function in osteoblasts and chondrocytes and probably be master control gene. The NRCAM gene is located on the chromosome 7q31, a region showed significant association with BMD in a published GWAS [35]. NRCAM is also a gene involved in regulation of estrogen. As one of the oocyte-specific genes, the NRCAM possess embryogenesis cellular growth and differentiation identified from the human primordial follicles cDNA library [36].

The IDS gene encodes Iduronate-2-sulfatase which is required for the lysosomal degradation of heparan sulfate and dermatan sulfate [37]. Mutations in the gene that result in enzymatic deficiency may lead to the sex-linked Mucopolysaccharidosis Type II [38]. Since glycosaminoglycans are fundamental in connective tissue structure and function, mucopolysaccharidosis disorders are characterized by severe skeletal abnormality including abnormal bone structure, growth failure and severe articular cartilage and joint problems [39]. The IDS gene also has relationship to AAM. IDS plays an important role in metabolism of steroid hormones such as estrone and estradiol [40]. Significant declines in activity of IDS was observed in the mammary cell lines MCF7 and T47D with estrogen exposure, with higher doses of estradiol associated with more significant declines [41]. The collective evidence suggests that IDS is important for both bone metabolism and AAM.

The LOC148145 gene locates on the chromosome 19q12. It is the non-coding RNA gene. The biological function of this gene is currently unclear and thus the exact mechanisms by which LOC148145 is involved in co-regulation of bone and AAM await discovery.

For the six SNPs associated with FNGPs and AAM, the effect directions were not completely consistent in the discovery and replication studies. The inconsistency could be explained by several reasons. First, it may be caused by genetic heterogeneity. For instance, allele frequencies of genetic variants could be different among diverse populations due to different evolution histories, which results in different genotype phenotype associations [42]. Recent studies showed that replicable findings in specific populations might be more generalizable in other populations, and such variants are more likely to be causal in nature [43]. Second, GWAS is an indirect association method based on linkage disequilibrium between SNP markers. Significant associations may be found at genetic markers that are in linkage disequilibrium with causal variants, rather than the causal variants *per se*. Therefore, inconsistency of the effect directions could be a result of different patterns of linkage disequilibrium in different populations.

Interestingly, there was an overlap between the results of this study and those of some published studies. Elks et al. reported the largest meta-analysis of GWAS for AAM in 87,802 women of European ancestry [44]. In that study, the SNP rs6589964 was strongly associated with AAM (*p* = 1.9×10^−12^). In our study, this SNP achieved *p* values of 6.64×10^−4^ to 8.79×10^−4^ for three trait pairs in the Caucasian discovery sample and p values of 2.86×10^−3^ to 6.27×10^−3^ in the Chinese replication sample.

In the current study, FNGPs were calculated based on DXA-derived FN BMD and bone size. This is a convenient method to obtain bone geometric indices using the areal BMD with the assumption that the mineral in the cross section is confined to an annular cortical region. However, the DXA measured BMD is restricted to two dimensions, and the resolution and accuracy of the structural parameters are affected. Despite this, studies showed that the geometry of femoral neck cross sections was reasonably well characterized by DXA compares to a more rigorous 3D finite element technique [45]. In particular, due to the wide availability of DXA scanners and the low radiation exposure of scanning, DXA is still the most popular method in clinical settings and bone research.

In summary, by performing a bivariate GWAS, we identified three novel genes (NRCAM, IDS and LOC148145) that may co-regulate FNGPs and AAM. Our findings need to be validated in different populations and molecular functional studies. Once confirmed, our findings may help improve our understanding of genetic architecture and pathophysiological mechanisms underlying osteoporosis and fracture risk. Our findings also furnish a foundation for further molecular and functional analyses of the genes in regulating timing of menarche and women’s health in general.
